# Early Versus Late Initiation of Renal Replacement Therapy Impacts Early Mortality in Diabetic Patients with Acute Kidney Injury After Cardiac Surgery

**DOI:** 10.3390/jcdd13090404

**Published:** 2026-08-22

**Authors:** Ozkan Ozler, Vedat Erentug

**Affiliations:** Department of Cardiovascular Surgery, Istanbul Mehmet Akif Ersoy Thoracic and Cardiovascular Surgery Training and Research Hospital, Istanbul 34303, Türkiye; drvedat2002@yahoo.com

**Keywords:** acute kidney injury, cardiac surgery, diabetes mellitus, renal replacement therapy

## Abstract

There are limited data on the optimal time of renal replacement therapy (RRT) after cardiac surgery, and published results are controversial. In our study, an answer was sought to the question of the correct timing of RRT in diabetic patients with post-cardiac surgery acute kidney injury (AKI). Forty-four adult patients with DM who required postoperative RRT were included in our single-center prospective study. According to the time of onset of RRT, the patients were divided into two groups: those whose RRT was initiated with the early protocol (RRT started before conventional urgent indications developed) or the standard protocol (RRT started after at least one conventional urgent indication developed). The primary endpoint was in-hospital mortality; secondary endpoints were RRT duration, renal recovery, and ICU and hospital length of stay. A total of 54.5% of the 44 participants were female, while the mean age was 65 ± 9. Early RRT protocol was performed on 21(47.7%) and the standard protocol was performed on 23 (52.3%) patients. Twenty-three patients (52.3%) died during follow-up. Patient survival was found to be significantly higher in the group of patients who received the early RRT protocol, based on univariate analysis, binary regression analysis, Cox regression analysis, and log-rank analysis, compared to the standard protocol group (respectively, *p* < 0.001, *p* < 0.03, *p* < 0.01, and *p* < 0.001). It was found that RRT initiation with an early protocol was advantageous in terms of survival in the post-cardiac surgery AKI group of diabetic patients. We believe that the study results provide guidance on the follow-up of this group of patients.

## 1. Introduction

The most common cause of acute kidney injury in intensive care units (ICUs) after sepsis is post-cardiac surgery acute kidney injury (AKI) [1,2]. The incidence of post-cardiac surgery AKI ranges between 7% and 40% [3]. In a study, it was found that the rate of AKI requiring renal replacement therapy (RRT) was 2.3% [4]. Post-cardiac surgery AKI is one of the major complications that affects mortality and morbidity [5]. Studies have shown that post-cardiac surgery AKI increases perioperative mortality by 3–8 times, and the mortality rate in patients with AKI ranges from 21.8% to 64.3% [6]. 

Renal replacement therapy should be started rapidly in cases of acute kidney injury-related and treatment-resistant hypervolemia, metabolic disorders, or electrolyte disorders. Cardiopulmonary bypass (CPB) contributes to renal injury through nonpulsatile flow, renal hypoperfusion, hemodilution, hemolysis, systemic inflammation, oxidative stress, microembolization, and ischemia–reperfusion injury. There is no clear data on the optimal time to initiate RRT in patients who develop AKI following cardiac surgery. In a multicenter, randomized controlled study, The Artificial Kidney Initiation in Kidney Injury (AKIKI) study, which monitored 620 AKI patients who developed stage 3 AKI in the ICU in 2016, no significant difference in mortality that developed within 90 days was found between the patient group that received early RRT and the patient group that received late RRT [7]. In a single-center study on early versus late initiation of renal replacement therapy in critically ill patients with acute kidney injury (ELAIN), the positive effect of early-onset RRT on life expectancy could be observed [8]. In these studies, the etiology of AKI was different. In the ELAIN study, about 50% of the patients included in the study were patients who underwent cardiac surgery. Therefore, the results of the ELAIN study suggest that early RRT may have a positive effect on patients who underwent cardiac surgery. In a retrospective observational study, which consisted of 203 patients, it was found that the patients who began receiving RRT after the 3rd day after cardiac surgery had higher hospital mortality and a longer hospital stay [9]. 

Diabetes mellitus (DM) affects 8.4% of the world’s population [10]. Diabetes-related vascular complications are the cause of the majority of disease-induced morbidity and mortality. While the prevalence of DM in patients undergoing cardiac surgery in the Western population is around 20%, it rises to 46.7% in the Asian population as a result of the higher number of patients with DM diagnosis [11]. Diabetes is a major risk factor for coronary artery disease and has a negative impact on cardiac surgery prognosis [11,12,13]. DM has been identified as an independent risk factor for the development of post-cardiac surgery AKI, the need for new RRT, and poor renal survival in previous studies [14]. Potential mechanisms include diabetic microvascular disease, endothelial dysfunction, impaired renal autoregulation, chronic inflammation and oxidative stress, and hyperglycemia-amplified ischemia–reperfusion injury. These abnormalities may reduce renal reserve during CPB and postoperative hemodynamic instability.

Our study aimed to investigate the effect of RRT initiation time on mortality in patients with DM, who constitute the majority of cardiac surgery patients, and in patients with post-cardiac surgery AKI. In addition, the mortality rate of patients with DM who underwent cardiac surgery and began receiving RRT was revealed. Our study provides guidance for the initiation of RRT in this patient group after AKI development.

## 2. Materials and Methods

### 2.1. Study Design and Patient Selection 

Forty-four adult patients in our clinic with type 2 diabetes who underwent open-heart surgery and required RRT in the postoperative period were included in our single-center prospective study. The study included patients over the age of 18, with type 2 DM, who developed AKI in the postoperative period after cardiac surgery and started to receive RRT. The study exclusion criteria are as follows: patients who received RRT in the preoperative period; died within 48 h of the postoperative period; received follow up with a stage 5 chronic renal failure diagnosis; developed AKI before surgery; were pregnant; had chronic liver disease; or had type 1 DM. Because the cohort was restricted to patients who received RRT, the study did not include a comparator group of patients with similar postoperative AKI who recovered without RRT. This study was conducted in accordance with the Declaration of Helsinki and informed written consent was obtained from each patient. The Istanbul Mehmet Akif Ersoy Thoracic and Cardiovascular Surgery Training and Research Hospital Institutional Review Board approved this study. Institutional Review Board Number 2020-11; date of approval 18 February 2020.

### 2.2. Data Collection and Outcome

The patients in our study were classified according to the clinical indication present at the time RRT was initiated. The standard indication group included patients in whom at least one conventional urgent indication for RRT had developed: treatment-resistant fluid overload despite appropriate medical and diuretic therapy; severe hyperkalemia (serum potassium > 6.0 mEq/L) refractory to medical treatment; a uremic complication, such as pericarditis or encephalopathy; or refractory metabolic acidosis (arterial pH ≤ 7.10 despite appropriate resuscitation and medical treatment).

The early RRT decision was made in accordance with the nephrologist’s decision. Patients in this group were those whose renal functions were not expected to improve in the near term and who were expected to progress in the near term, although conventional urgent indications had not yet been developed. The assessment was based on the overall postoperative clinical course, including serial renal function measurements, urine output trends, volume status, acid–base and electrolyte trends, and hemodynamic condition. Intermittent hemodialysis (IHD) and continuous renal replacement therapy (CRRT) options were also preferred for RRT in line with the nephrologist’s decision. Using the Seldinger technique for vascular access, a double-lumen hemodialysis catheter was inserted into the internal jugular vein or femoral vein. As anticoagulants, heparin and low molecular weight heparin were used, or dialysis without anticoagulant was performed in line with the clinician’s decision. 

In the preoperative period, patients’ age at admission and clinical, demographic, and laboratory parameters were recorded. Estimated glomerular filtration rate (eGFR) was calculated using the CKD-EPI [Chronic Kidney Disease Epidemiology Collaboration] formula [15]. There are several models available to determine the AKI risk in patients who will undergo cardiac surgery. The Thakar Score (Cleveland Clinic Score) and the Mehta Score are the two most sensitive scoring systems for predicting the severe AKI risk requiring dialysis [16,17]. The Thakar Score is one step ahead of the Mehta Score [18]. The Thakar Score was used in our study. Score parameters and scoring are as stated: female sex, 1 point; congestive heart failure, 1 point; left ventricular ejection fraction (EF) < 35%, 1 point; preoperative use of intra-aortic balloon pump (IABP), 2 points; chronic obstructive pulmonary disease, 1 point; DM requiring insulin, 1 point; previous heart surgery, 1 point; emergency surgery, 2 points; type of surgery being coronary artery bypass graft (CABG), 0 points; valve surgery, 1 point; valve surgery with CABG, 2 points; other heart surgeries, 2 points; and preoperative creatinine: if between 1.2 and 2.1 mg/dL, 2 points, if ≥2.1 mg/dL, 5 points. Estimated dialysis risk percentages based on risk scores are as follows: 0–2 points 0.4%; 3–5 points 1.8%; 6–8 points 7.8–9.5%; and 9–13 points 21.5%. 

The presence of any of the following criteria were accepted for the diagnosis of diabetes mellitus; fasting blood glucose > 126 mg/dL; HbA1c ≥ 6.5%; blood glucose above 200 mg/dL at any time of the day with hyperglycemia symptoms; and blood glucose ≥ 200 mg/dL after the 2nd hour of the 75 g oral glucose tolerance test [19]. Antihyperlipidemic therapy or LDL cholesterol > 70 mg/dL was the accepted criteria for hyperlipidemia diagnosis. No longer requiring RRT was considered the criterion for kidney recovery time. 

The difference in hospital mortality between patients with early and late RRT was the primary endpoint of our study. RRT duration, ICU and hospital stay, and improvement in renal function in patients who began to receive early and late RRT were secondary endpoints.

### 2.3. Statistical Analysis

The effect size for mortality of the study and control groups was found to be 0.38 (alpha error probability = 0.05) in the power analysis of our study performed with the G*power 3.1 program. In the sample size analysis performed by taking the power value of 0.80, the total number of samples to be taken was found to be 40 (20 patients for each group). In this study, statistical analyses were performed with the NCSS (Number Cruncher Statistical System) 2007 Statistical Software (UT, USA) package program. 

To compare clinical and laboratory characteristics, the Student’s *t*-test was used if the data were normally distributed, and the Mann–Whitney U test was used if otherwise. The differences between categorical variables were evaluated using the Chi-square test and Fisher’s exact test. Binary logistic regression analysis was performed to determine the effective factors on mortality. The log-rank test was used to compare the survival curves between the groups in which RRT was started early or with standard criteria. Cox regression analysis was performed to determine the factors affecting patient survival time. The results’ statistical significance level was determined based on a *p*-value of <0.05.

## 3. Results

Our study included 44 patients with type 2 DM who needed RRT in the postoperative period. A total of 54.5% of the participants were female, while the mean age of the patients was 65 ± 9. The mean EF was 46 ± 11% and the chronic kidney disease (stage 3 and stage 4) incidence was 50%. Early RRT protocol was performed on 21 (47.7%) and the standard protocol was performed on 23 (52.3%) of the patients in the RRT initiation. A total of 23 (52.3%) of the patients died. The survival rate was 47%. At the time of discharge, only one of the 21 surviving patients required dialysis. More than half of the patients had undergone heart valve surgery. Approximately 25% of the patients included in the study were oligoanuric in the pre-RRT period. The median value of urine output in the last 1 h was 20 cc. The CRRT regimen was adopted for approximately 70% of patients. Table 1 shows the patients’ demographic information, preoperative laboratory parameters, operation-related factors, postoperative period data, and survival analysis results. 

There were no significant differences between the early and standard protocol groups in terms of gender, age, comorbid diseases analyzed (chronic kidney disease, chronic obstructive pulmonary disease, hypertension, and hyperlipidemia), laboratory parameters, type of operation, duration of cardiopulmonary bypass, and cross-clamp time. The analysis results of the relationship between preoperative laboratory data, demographic characteristics, patients’ intraoperative and postoperative period data, and the patient groups in which RRT was initiated using the early protocol versus the patient groups in which RRT was initiated using the standard protocol are given in Table 2. 

**Figure 1:**
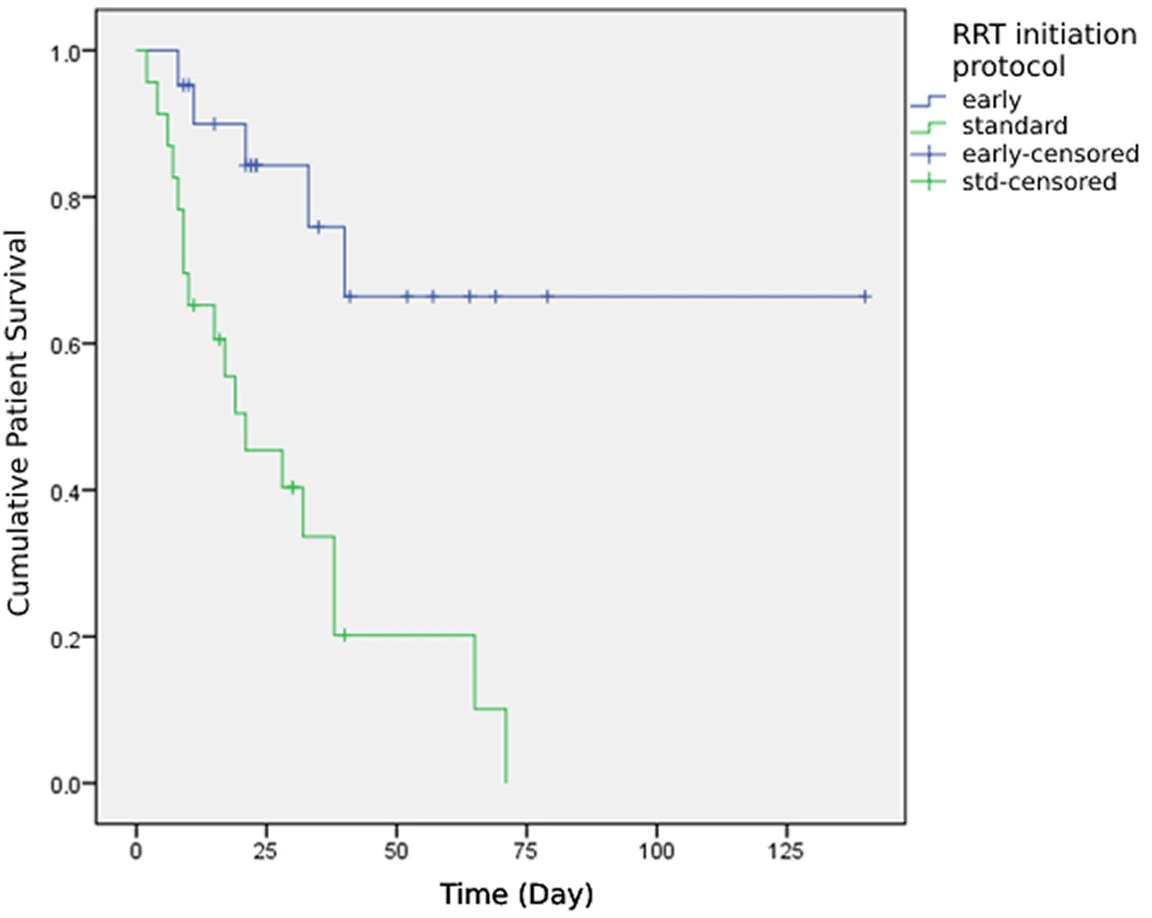
RRT relationship between the RRT initiation time protocol and patient survival time, log-rank analysis (*p* < 0.001).

Patient survival was found to be higher in the group of patients who began receiving RRT with the early protocol compared to the group who began receiving RRT with the standard protocol (*p* < 0.001). In deceased patients, the myocardial infarction (MI) history rate was significantly higher than in surviving patients. When RRT was initiated, a negative correlation was found between pH and low bicarbonate, elevated lactate, and survival. It was found that the survival rate decreased with the prolongation of cardiopulmonary bypass time. The relationship between preoperative laboratory data, demographic characteristics, intraoperative parameters, postoperative data, and survival are shown in Table 3. Binary logistic regression analysis was performed with variables found to be significant in single variable statistical analysis. With this analysis, early initiation of the RRT protocol was found to be an effective parameter in patient survival (Table 3). The relationship between the RRT initiation protocol and patient survival is also shown in the log-rank analysis in Figure 1 (*p* < 0.001).

Smoking, previous MI, blood pH in the pre-RRT period, blood lactate level in the pre-RRT period, and RRT initiation protocol were found to be significant in terms of patient survival (Table 3).

## 4. Discussion

Although the mortality rate after cardiac surgery without acute kidney injury ranges between 1% and 8% [20], the rate increases in patients with AKI [21]. In patients who developed AKI and needed RRT, on the other hand, it has been associated with an increase in mortality of up to 63% [22]. There are a limited number of randomized controlled trials (RCTs) on absolute renal replacement therapy (RRT) indications for its initiation (resistant electrolyte and metabolic disorders, resistant hypervolemia, and presence of uremic symptoms). Moreover, there are a limited number of RCTs on the effect of starting RRT before complications of renal failure or the development of renal failure on mortality. The answer to whether possible metabolic disorders will affect arrhythmia and myocardial performance more easily, and whether they can partially fulfill compensatory mechanisms against the volume load that may occur, is especially important for patients who develop AKI after cardiac surgery. Diabetic patients, on the other hand, constitute the fragile patient group, which is known to have multiple vascular systems affected and weakened autonomic reflex mechanisms and neurohumoral response in the changing metabolic state of the body. Our study shows the effect of early initiation of RRT on mortality in patients with DM who develop post-cardiac surgery AKI, which constitutes the vast majority of patients undergoing cardiac surgery.

Although there are no RCTs regarding renal replacement therapy initiation time in the diabetic patient group developing post-cardiac surgery AKI, there are two large prospective RCTs involving postoperative patients followed in the general ICU. In the ELAIN study [8], in which 216 patients participated, it was shown that the 90-day mortality was lower in the group that started RRT with the early protocol. In the AKIKI study [7], which included 620 patients, no significant difference was found between the two groups in terms of 60-day mortality. Although both AKIKI and ELAIN are major studies on the subject, they were conducted in general ICU patients. Therefore, it cannot be expected that the results will be fully applicable to patients who develop AKI following cardiac surgery. It is clear, however, that the fact that approximately half of the patients included in the ELAIN study—in which mortality was reduced with early-initiated RRT—were patients who received follow up in the ICU after cardiac surgery, provides more supportive information about when to start RRT after cardiac surgery. Approximately 20% of patients in the AKIKI study began RRT following cardiac surgery. In the AKIKI study, it was found that 50% of the patients who met the criteria for early RRT but were randomized to the patient group who started RRT with the standard protocol did not require RRT. Therefore, it is thought that it would be more rational to start RRT before life-threatening complications develop in patients with predicted renal function progression. This can be achieved by including blood or urine markers in our routine practice; these markers increase earlier than creatinine when measured in urine and blood, providing information about the progression of renal damage. RCTs including these markers are needed for the patient group who developed post-cardiac surgery AKI and began to receive RRT. In a review that included two RCTs and nine observational studies on patients who had undergone cardiac surgery, which included 847 patients, it was shown that 28-day mortality was reduced by early RRT [23] (*p* < 0.0001). 

In many studies, the group that received early RRT after cardiac surgery appeared to be advantageous in terms of mortality [24,25]. In our study, it is noteworthy that the mortality rate is reduced by 78.3 to 23.8 percent in the early RRT group. This difference may be more remarkable in diabetic patients due to acidosis and a reduced ability to adapt to changes in the metabolic image (electrolyte, pH, etc.) in this patient group. Apart from macrovascular complications, the prevalence of microvascular disease in this patient group, as well as the weakening of neurohumoral responses due to autonomic neuropathy, could explain this remarkable difference. Another mechanism that may be effective in the physiopathology of early RRT’s beneficial effect on mortality is as follows: Early removal of the volume load reduces the load on the heart, improving its performance and preserving perfusion of the kidney and other vital organs. As expected, compensatory mechanisms against volume load were weakened in the diabetic group, whose cardiac performance was decreased even in the preoperative period in the majority of patients. If the volume load is not removed early, the increased oxygen requirement of the myocardium may result in myocardial ischemia with type 2 myocyte damage in the early period. Furthermore, it may play a role in mortality by affecting kidney perfusion and vital organs due to decreased myocardial performance. Arrhythmogenic conditions due to cardiac depression or electrolyte disorders due to acidosis can be prevented with early RRT. 

When we reviewed the studies in the literature, we discovered that the mortality rate in the patient group requiring postop RRT after cardiac surgery was in the range of 60–70% [26,27,28,29,30,31]. In the study by Xiao-mei Yang et al., which included non-DM patients as well, in-hospital mortality was found to be 46.8% in all patients, 33.9% in the early RRT group, and 51.9% in the group that received RRT with the standard protocol, respectively [32]. Although there is insufficient data on mortality in diabetic patients who began receiving RRT in the postoperative period, the results are thought to be similar considering that in-hospital mortality was found to be increased in DM patients after cardiac surgery in the study of J. Bucerius et al. [11].

In a retrospective study conducted in 2013, none of the 70 surviving patients out of 107 who began receiving RRT after cardiac surgery required RRT at discharge [33]. In their study, Thongprayoon C. et al. found that 8 (4%) of 186 patients who developed post-cardiac surgery AKI and required RRT continued to require RRT at the end of the first year [34]. In our study, one of the surviving patients (4.7%) required RRT after discharge; this patient had a preoperative diagnosis of stage 4 CKD and was in the group that received RRT with the standard protocol.

In our study, univariate analysis revealed that survival in the IHD group was superior to that in the CRRT group. No randomization was performed for IHD and CRRT for renal replacement therapy at the beginning of the study. The type of RRT appropriate for the patient’s hemodynamic and clinical condition was preferred. Therefore, the significance discovered in univariate analyses in the IHD group was attributed to the initiation of CRRT in the hemodynamically unstable group, which was at a higher risk for survival. As expected, blood pH and bicarbonate levels were higher, and creatinine levels were lower in the early-initiated RRT protocol.

## 5. Conclusions

In our study, we discovered that initiating RRT before the development of conventional urgent indications positively affected early survival in diabetic patients with post-cardiac surgery AKI. Our study is considered a guide for the decision to initiate RRT in diabetic patients after cardiac surgery. 

## Figures and Tables

**Table 1:** Demographic data, preoperative laboratory parameters, operation-related factors, postoperative period data, and survival analysis results of the patients.

Demographic Data and Preoperative Laboratory Parameters of the Patients	
	Mean ± SD
Female, n (%)	24 (54.5)
Age (years)	65 ± 9
Body mass index (kg/m^2^)	29.2 ± 5.4
Insulin therapy, n (%)	20 (44.5)
Hypertension, n (%)	25 (56.8)
COPD, n (%)	8 (18.2)
Hyperlipidemia, n (%)	29 (65.9)
Prior cardiac surgery, n (%)	3 (6.8)
Prior myocardial infarction, n (%)	15 (34.1)
Preoperative ejection fraction (%)	46 ± 11
CKD at admission, n (%)	22 (50)
Preoperative:	
BUN (mg/dL)	28 ± 13
Creatinine (mg/dL)	1.2 ± 0.6
eGFR (mL/min/1.73 m^2^)	63.5 ± 26.3
Sodium (mEq/L)	137 ± 3
Potassium (mEq/L)	4.4 ± 0.5
Hemoglobin (gr/dL)	10.8 ± 1.5
HbA1c (%)	7.8 ± 1.4
CRP (mg/L), median (IQR)	5.3 (IQR 2.4–9.5)
Total cholesterol (mg/dL)	179 ± 49
LDL cholesterol(mg/dL)	106 ± 41
HDL cholesterol (mg/dL)	39 ± 13
Triglyceride (mg/dL)	161 ± 69
Proteinuria, n (%)	23 (53.7)
Thakar score (%), median (IQR)	1.8 (IQR 1.8–9.5)
Preoperative IABP, n (%)	0
Operation-related factors:	
	n (%)
Cardiopulmonary bypass time (minute), mean ± SD	135 ± 55
Cross-clamp time (minutes), mean ± SD	77 ± 40
Intraoperative IABP	1 (2.3)
Urgency of the operation	6 (13.6)
The operation performed:	
CABG	17 (38.6)
CABG + heart valve surgery	15 (34.1)
Heart valve surgery	9 (20.5)
Other	3 (6.8)
Postoperative period data:	
	Mean ± SD
Postoperative ECMO, n (%)	2 (4.5)
Postoperative IABP, n (%)	4 (9.1)
Postoperative inotropic support, n (%)	29 (65.9)
Re-operation, n (%)	19 (43.2)
Time between AKI and onset of RRT (days), median (IQR)	1 (0–2)
Time between operation and RRT (days), median (IQR)	3 (1–5)
Before RRT:	
Last 1 h of urine output (cc), median (IQR)	20 (0–30)
Last 6 h of total urine output (cc), median (IQR)	100 (27–267)
Total urine output in the last 12 h (cc)	447 ± 396
Total urine output in the last 24 h (cc)	1166 ± 775
Oliguria	8 (19)
Anuria	2 (4.8)
At the start of the RRT:	
BUN (mg/dL)	56 ± 25
Creatinine (mg/dL)	2.8 ± 1.2
pH	7.28 ± 0.12
Bicarbonate (mEq/L)	17.2 ± 3.8
Potassium (mEq/L)	4.6 ± 0.8
pCO_2_ (mmHg)	34.6 ± 6.6
Lactate, median (IQR)	2.1 (1.3–6.0)
RRT type, n (%):	
CRRT	31 (70.5)
IHD	7 (15.9)
IHD + CRRT	6 (13.6)
RRT protocol, n (%):	
Early	21 (47.7)
Standard	23 (52.3)
RRT treatment, length of hospital stay and survival analysis results of the patients:	
	Mean ± SD
RRT duration (gün), median (IQR)	3 (IQR 2–4.5)
RRT duration of surviving patients (days), median (IQR)	2 (IQR 2–3)
RRT duration of deceased patients (days), median (IQR)	3 (IQR 1.5–6.5)
Discharge:	
BUN (mg/dL)	33 ± 17
Creatinine (mg/dL)	1.45 ± 0.96
eGFR (mL/min/1.73 m^2^)	53 ± 23
Sodium (mEq/L)	134.9 ± 3.6
Potassium (mEq/L)	4.7 ± 0.5
Length of hospital stay (day)	30 ± 26.2
ICU length of stay (day)	18.3 ± 16.4
Need for dialysis at discharge, n (%)	1 (4.8)
Patient survival (death), n (%)	23 (52.3)

COPD: chronic obstructive pulmonary disease, EF: ejection fraction; CKD: chronic kidney disease; eGFR: estimated glomerular filtration rate; BUN: blood urea nitrogen; CRP: C-reactive protein; LDL: low density lipoprotein; HDL: high density lipoprotein; IABP: intra-aortic balloon pump; CABG: coronary artery bypass graft; AKI: acute kidney injury; ECMO: extracorporeal membrane oxygenation; RRT: renal replacement therapy; CRRT: continuous renal replacement therapy; IHD: intermittent hemodialysis; ICU: intensive care unit; IQR: interquartile range; and mean ± SD: mean ± standard deviation.

**Table 2:** The relationship between preoperative laboratory data, demographic data, and intraoperative and postoperative period data of the patients and the patient groups in whom RRT was initiated with early and standard protocols.

**The relationship between the preoperative laboratory data and demographic characteristics of the patients and the RRT initiation protocol (early–standard) (the data are given as mean ± standard deviation).**			
	Early Protocol (n = 21)	Standard Protocol (n = 23)	*p*
Female, n (%)	12 (57.1)	12 (52.2)	0.74
Age (years)	65.2 ± 8.7	64.7 ± 9.5	0.84
Body mass index (kg/m^2^)	28.5 ± 5.4	29.8 ± 5.2	0.43
Insulin therapy, n (%)	10 (47.6)	10 (43.5)	0.78
Hypertension, n (%)	11 (52.4)	14 (60.9)	0.57
COPD, n (%)	3 (14.3)	5 (21.7)	0.52
Hyperlipidemia, n (%)	13 (60.9)	16 (69.6)	0.59
Prior open-heart surgery, n (%)	0	3 (13)	0.23
Prior myocardial infarction, n (%)	5 (23.8)	10 (43.5)	0.23
Preoperative ejection fraction (%)	43.5 ± 11.9	49.4 ± 11.5	0.1
CKD at admission, n (%)	11 (52.4)	11 (47.8)	0.76
Preoperative:			
BUN (mg/dL)	29 ± 12.1	27.7 ± 14	0.76
Creatinine (mg/dL)	1.2 ± 0.7	1.1 ± 0.6	0.7
eGFR (mL/min/1.73 m^2^)	61.3 ± 28.1	65.5 ± 25	0.6
Sodium (mEq/L)	136.7 ± 3.3	138 ± 3.3	0.054
Potassium (mEq/L)	4.4 ± 0.4	4.4 ± 0.5	0.81
Hemoglobin (gr/dL)	10.8 ± 1.3	11 ± 1.6	0.63
HbA1c (%)	8.1 ± 1.6	7.3 ± 1	0.055
CRP (mg/L), median (IQR)	4.7 (2.4–13.7)	5.8 (2.4–8.8)	0.9
Total cholesterol (mg/dL)	165 ± 46	193 ± 50	0.08
LDL cholesterol (mg/dL)	98 ± 41	114 ± 41	0.23
HDL cholesterol (mg/dL)	37 ± 12	42 ± 13	0.25
Triglyceride (mg/dL)	145 ± 65	176 ± 70	0.19
Proteinuria, n (%)	10 (58.8)	13 (54)	0.76
Thakar score (%), median (IQR)	1.8 (1.8–9.5)	1.8(1.8–1.8)	0.39
Preoperative IABP, n (%)	0	0	
**The relationship between intraoperative parameters, urgency of the operation, and the operation performed and the patient groups in whom RRT was started with the early and standard protocols (the data are given as n (%)).**			
	Early protocol (n = 21)	Standard protocol (n = 23)	*p*
Cardiopulmonary bypass time (minute), mean ± SD	124 ± 45	146 ± 62	0.19
Cross-clamp time (minutes), mean ± SD	72 ± 33	81 ± 46	0.43
Intraoperative IABP	0	1 (4.3)	1
Urgency of the operation	3 (14.3)	3 (13.3)	0.62
The operation performed:			
CABG	8 (38.1)	9 (39.1)	0.92
CABG + heart valve surgery	7 (33.3)	8 (34.8)	
Heart valve surgery	4 (19)	5 (21.7)	
Other	2 (9.5)	1 (4.3)	
**The relationship between laboratory parameters, urine output levels, RRT type, and the parameters related to hemodynamic status before starting RRT in the postoperative period and RRT initiation protocols (the data are given as mean ± standard deviation).**			
	Early protocol (n = 21)	Standard protocol (n = 23)	*p*
Postoperative ECMO, n (%)	0	2 (8.7)	0.48
Postoperative IABP, n (%)	2 (9.5)	2 (8.7)	1
Postoperative inotropic support, n (%)	11 (52.4)	18 (78.3)	0.07
Re-operation, n (%)	5 (23.8)	14 (60.9)	0.03
Time between AKI and onset of RRT (days), median (IQR)	0 (0–1)	1 (0–2)	0.02
Time between operation and RRT (days), median (IQR)	2 (1–4)	3 (2–8)	0.21
Before RRT:			
Last 1 h of urine output (cc), median (IQR)	20 (5–30)	15 (0–35)	0.54
Last 6 h of total urine output (cc), median (IQR)	100 (60–237)	100 (7.5–310)	0.67
Total urine output in the last 12 h (cc)	473 ± 401	424 ± 398	0.69
Total urine output in the last 24 h (cc)	1318 ± 785	1027 ± 757	0.23
Oliguria	4 (20)	4 (18.2)	0.88
Anuria	0	2 (9.1)	0.48
At the start of the RRT:			
BUN (mg/dL)	50 ± 16	62 ± 30	0.12
Creatinine (mg/dL)	2.3 ± 0.9	3.2 ± 1.2	0.02
pH	7.32 ± 0.05	7.25 ± 0.15	0.07
Bicarbonate (mEq/L)	18.4 ± 3	16.2 ± 4.2	0.06
Potassium (mEq/L)	4.6 ± 0.7	4.7 ± 0.8	0.8
Lactate	2.5 ± 1.8	4.6 ± 4.1	0.04
pCO_2_ (mmHg)	35 ± 5	33 ± 7	0.37
RRT type, n (%):			
CRRT	13 (61.9)	18 (78.3)	0.46
IHD	4 (19)	3 (13)	
IHD + CRRT	4 (19)	2 (8.7)	
**Relationship between RRT protocol and survival, discharge laboratory values, and length of hospital stay (the data are given as mean ± standard deviation).**			
	Early protocol (n = 21)	Standard protocol (n = 23)	*p*
RRT duration of surviving patients (days), median (IQR)	2 (1.25–3.75)	2.5 (2–3)	0.69
Need for dialysis at discharge, n (%)	0	1 (20)	0.23
Discharge:			
BUN (mg/dL)	34 ± 15.1	32 ± 22	0.87
Creatinine (mg/dL)	1.4 ± 0.9	1.5 ± 1.1	0.91
eGFR (mL/min/1.73 m^2^)	54 ± 25	51 ± 20	0.79
Sodium (mEq/L)	135 ± 3	134 ± 3	0.81
Potassium (mEq/L)	4.6 ± 0.5	4.9 ± 0.4	0.26
Length of hospital stay (day)	37.8 ± 31.3	22.8 ± 18.4	0.06
ICU length of stay (day)	18.2 ± 15.3	18.4 ± 17.7	0.97
Patient survival (death), n (%)	5 (23.8)	18 (78.3)	<0.001

COPD: chronic obstructive pulmonary disease; EF: ejection fraction; CKD: chronic kidney disease; eGFR: estimated glomerular filtration rate; BUN: blood urea nitrogen; CRP: C-reactive protein; LDL: low density lipoprotein; HDL: high density lipoprotein; IABP: intra-aortic balloon pump; CABG: coronary artery bypass graft; AKI: acute kidney injury; ECMO: extracorporeal membrane oxygenation; RRT: renal replacement therapy; CRRT: continuous renal replacement therapy; IHD: intermittent hemodialysis; ICU: intensive care unit; IQR: interquartile range; and mean ± SD: mean ± standard deviation.

**Table 3:** The relationship between preoperative laboratory data, demographic characteristics, intraoperative parameters, postoperative period data, and the survival of patients.

**The relationship between preoperative laboratory data, demographic characteristics, and survival of the patients (the data are given as mean ± standard deviation).**			
	Deceased (n = 23)	Alive (n = 21)	*p*
Female, n (%)	11 (47.8)	13 (61.9)	0.34
Age (years)	64.7 ± 9.1	65.3 ± 9.2	0.81
Body mass index (kg/m^2^)	29.1 ± 4.8	29.3 ± 5.9	0.87
Insulin therapy, n (%)	10 (43.5)	10 (47.6)	0.78
Hypertension, n (%)	14 (60.9)	11 (52.4)	0.57
COPD, n (%)	5 (21.7)	3 (14.3)	0.7
Hyperlipidemia, n (%)	16 (69.6)	13 (61.9)	0.59
Prior open-heart surgery, n (%)	3 (13)	0	0.08
Prior myocardial infarction, n (%)	11 (47.8)	4 (19)	0.04
Preoperative ejection fraction (%)	47 ± 12	45 ± 11	0.63
CKD at admission, n (%)	9 (39.1)	13 (61.9)	0.13
Preoperative:			
BUN (mg/dL)	28 ± 14	28 ± 11	0.9
Creatinine (mg/dL)	1.1 ± 0.6	1.3 ± 0.7	0.56
eGFR (mL/min/1.73 m^2^)	67 ± 26	58 ± 26	0.26
Sodium (mEq/L)	138 ± 3	136 ± 2	0.11
Potassium (mEq/L)	4.4 ± 0.5	4.4 ± 0.4	0.94
Hemoglobin (gr/dL)	10.6 ± 1.5	11.2 ± 1.3	0.22
HbA1c (%)	7.5 ± 1.1	8 ± 1.5	0.29
CRP (mg/L), median (IQR)	4.4 (2.3–8.2)	7 (3.6–11.7)	0.13
Total cholesterol (mg/dL)	183 ± 51	174 ± 48	0.55
LDL cholesterol (mg/dL)	108 ± 40	104 ± 43	0.79
HDL cholesterol (mg/dL)	38 ± 9	40 ± 10	0.68
Triglyceride (mg/dL)	174 ± 77	145 ± 56	0.2
Proteinuria n (%)	12 (54.5)	11 (57.8)	0.82
Thakar score (%), median (IQR)	1.8 (1.8–1.8)	1.8 (0.4–9.5)	0.37
Preoperative IABP, n (%)	0	0	
**The relationship between intraoperative parameters, operative urgency, the operation performed, and survival (the data are given as n (%)).**			
	Deceased (n = 23)	Alive (n = 21)	*p*
Cardiopulmonary bypass time (minute), mean ± SD	153 ± 57	116 ± 46	0.02
Cross-clamp time (minutes), mean ± SD	85 ± 45	67 ± 33	0.13
Intraoperative IABP	1 (4.3)	0	0.33
Urgency of the operation	3 (13)	3 (14.3)	0.9
The operation performed:			
CABG	6 (26.1)	11 (52.4)	0.34
CABG + heart valve surgery	9 (39.1)	6 (28.6)	
Heart valve surgery	6 (26.1)	3 (14.3)	
Other	2 (8.7)	1 (4.8)	
**The relationship between laboratory parameters, urine output levels, RRT type, and hemodynamic status and parameters associated with survival before initiation of postoperative RRT (the data are given as mean ± standard deviation).**			
	Deceased (n = 23)	Alive (n = 21)	*p*
Postoperative ECMO, n (%)	2 (8.7)	0	0.16
Postoperative IABP, n (%)	2 (8.7)	2 (9.5)	0.92
Postoperative inotropic support, n (%)	15 (65.2)	14 (66.7)	0.91
Re-operation, n (%)	12 (52.2)	7 (33.3)	0.2
RRT protocol, n (%):			
Early protocol	5 (21.7)	15 (71.4)	<0.001
Standard protocol	18 (78.3)	6 (28.6)	
Time between AKI and onset of RRT (days), median (IQR)	1 (0–2)	1 (0–1)	0.94
Time between operation and RRT (days), median (IQR)	3 (1–8)	2 (1.5–4)	0.37
Before RRT:			
Last 1 h of urine output (cc), median (IQR)	15 (0–30)	20 (5–40)	0.47
6 h urine output (cc), median (IQR)	90 (0–265)	125 (62–282)	0.28
12 h urine output (cc)	456 ± 437	438 ± 356	0.88
24 h urine output (cc)	1247 ± 879	1076 ± 653	0.47
Oliguria, n (%)	4 (18.2)	4 (20)	0.88
Anuria, n (%)	2 (9.1)	0	0.16
At the start of the RRT:			
BUN (mg/dL)	55 ± 25	57 ± 25	0.79
Creatinine (mg/dL)	2.7 ± 1.3	2.9 ± 0.9	0.59
pH	7.23 ± 0.14	7.3 ± 0.06	0.008
Bicarbonate (mEq/L)	15.9 ± 4.3	18.6 ± 2.7	0.01
Potassium (mEq/L)	4.6 ± 0.8	4.6 ± 0.7	0.97
Lactate	5.2 ± 3.9	2.1 ± 1.5	0.003
pCO_2_ (mmHg)	34 ± 7	34 ± 5	0.86
RRT type, n (%):			
CRRT	18 (78.3)	13 (61.9)	0.002
IHD	1 (4.3)	6 (28.6)	
IHD + CRRT	4 (17.4)	2 (9.5)	
**The relationship between survival and hospital stay and the discharge laboratory data of surviving patients (the data are given as mean ± standard deviation).**			
	Deceased (n = 23)	Alive (n = 21)	*p*
RRT duration (days) of patients, median (IQR)	4 (2–9)	2 (2–3)	0.04
Need for dialysis at discharge, n (%)		1 (4.8)	
Discharge:			
BUN (mg/dL)		33 ± 17	
Creatinine (mg/dL)		1.4 ± 0.9	
eGFR (mL/min/1.73 m^2^)		53 ± 23	
Sodium (mEq/L)		134 ± 3	
Potassium (mEq/L)		4.7 ± 0.5	
Length of hospital stay (day)	22.2 ± 18.6	38.5 ± 30.8	0.04
ICU length of stay (day)	20.5 ± 18	15.9 ± 14.5	0.35

COPD: chronic obstructive pulmonary disease; BUN: blood urea nitrogen; LDL: low density lipoprotein; HDL: high density lipoprotein; EF: ejection fraction; CKD: chronic kidney disease; eGFR: estimated glomerular filtration rate; CRP: C-reactive protein; IABP: Intra-aortic balloon pump; CABG: coronary artery bypass graft; AKI: acute kidney injury; ECMO: extracorporeal membrane oxygenation; RRT: renal replacement therapy; CRRT: continuous renal replacement therapy; IHD: intermittent hemodialysis; ICU: intensive care unit; IQR: interquartile range; and mean ± SD: mean ± standard deviation.

## Data Availability

The original contributions presented in this study are included in the article. Further inquiries can be directed to the corresponding authors.

## Abbreviations

The following abbreviations are used in this manuscript:

AKI	Acute kidney injury
CKD	Chronic kidney disease
CKD-EPI	Chronic Kidney Disease Epidemiology Collaboration
CRRT	Continuous renal replacement therapy
CABG	Coronary artery bypass graft
DM	Diabetes mellitus
eGFR	Estimated glomerular filtration rate
EF	Ejection fraction
IABP	Intra-aortic balloon pump
ICU	Intensive care unit
IHD	Intermittent hemodialysis
MI	Myocardial infarction
RCTs	Randomized controlled trials
RRT	Renal replacement therapy

## References

[1] Myburgh J.A., Finfer S., Bellomo R., Billot L., Cass A., Gattas D., Glass P., Lipman J., Liu B., McArthur C. (2012). Hydroxyethyl starch or saline for fluid resuscitation in intensive care. N. Engl. J. Med..

[2] Uchino S., Kellum J.A., Bellomo R., Doig G.S., Morimatsu H., Morgera S., Schetz M., Tan I., Bouman C., Macedo E. (2005). Acute renal failure in critically ill patients: A multinational, multicenter study. JAMA.

[3] Machado M.N., Nakazone M.A., Maia L.N. (2014). Prognostic value of acute kidney injury after cardiac surgery according to kidney disease: Improving global outcomes definition and staging (KDIGO) criteria. PLoS ONE.

[4] Hu J., Chen R., Liu S., Yu X., Zou J., Ding X. (2016). Global Incidence and Outcomes of Adult Patients With Acute Kidney Injury After Cardiac Surgery: A Systematic Review and Meta-Analysis. J. Cardiothorac. Vasc. Anesth..

[5] Hoste E.A., Cruz D.N., Davenport A., Mehta R.L., Piccinni P., Tetta C., Viscovo G., Ronco C. (2008). The epidemiology of cardiac surgery-associated acute kidney injury. Int. J. Artif. Organs..

[6] Bellomo R., Ronco C., Mehta R.L., Asfar P., Boisrame-Helms J., Darmon M., Diehl J.L., Duranteau J., Hoste E.A.J., Olivier J.B. (2017). Acute kidney injury in the ICU: From injury to recovery: Reports from the 5th Paris International Conference. Ann. Intensive Care.

[7] Gaudry S., Hajage D., Schortgen F., Martin-Lefevre L., Pons B., Boulet E., Boyer A., Chevrel G., Lerolle N., Carpentier D. (2016). Initiation Strategies for Renal-Replacement Therapy in the Intensive Care Unit. N. Engl. J. Med..

[8] Zarbock A., Kellum J.A., Schmidt C., Van Aken H., Wempe C., Pavenstadt H., Boanta A., Gerß J., Meersch M. (2016). Effect of Early vs Delayed Initiation of Renal Replacement Therapy on Mortality in Critically Ill Patients With Acute Kidney Injury: The ELAIN Randomized Clinical Trial. JAMA.

[9] Garcia-Fernandez N., Perez-Valdivieso J.R., Bes-Rastrollo M., Vives M., Lavilla J., Herreros J., Monedero P., on behalf of the GEDRCC (Grupo Español de Disfunción Renal en Cirugía Cardiaca) (2011). Timing of renal replacement therapy after cardiac surgery: A retrospective multicenter Spanish cohort study. Blood Purif..

[10] Bank I.E.M., Gijsberts C.M., Teng T.K., Benson L., Sim D., Yeo P.S.D., Ong H.Y., Jaufeerally F., Leong G.K.T., Ling L.H. (2017). Prevalence and Clinical Significance of Diabetes in Asian Versus White Patients With Heart Failure. JACC Heart Fail..

[11] Bucerius J., Gummert J.F., Walther T., Doll N., Falk V., Onnasch J.F., Barten M.J., Mohr F.W. (2003). Impact of diabetes mellitus on cardiac surgery outcome. Thorac. Cardiovasc. Surg..

[12] Gum P.A., O’Keefe J.H., Borkon A.M., Spertus J.A., Bateman T.M., McGraw J.P., Sherwani K., Vacek J., McCallister B.D. (1997). Bypass surgery versus coronary angioplasty for revascularization of treated diabetic patients. Circulation.

[13] Morris J.J., Smith L.R., Jones R.H., Glower D.D., Morris P.B., Muhlbaier L.H., Reves J.G., Rankin J.S. (1991). Influence of diabetes and mammary artery grafting on survival after coronary bypass. Circulation.

[14] Girman C.J., Kou T.D., Brodovicz K., Alexander C.M., O’Neill E.A., Engel S., Williams-Herman D.E., Katz L. (2012). Risk of acute renal failure in patients with Type 2 diabetes mellitus. Diabet. Med..

[15] Levey A.S., Stevens L.A., Schmid C.H., Zhang Y.L., Castro A.F., Feldman H.I., Kusek J.W., Eggers P., Van Lente F., Greene T. (2009). A new equation to estimate glomerular filtration rate. Ann. Intern. Med..

[16] Thakar C.V., Arrigain S., Worley S., Yared J.P., Paganini E.P. (2005). A clinical score to predict acute renal failure after cardiac surgery. J. Am. Soc. Nephrol..

[17] Mehta R.H., Grab J.D., O’Brien S.M., Bridges C.R., Gammie J.S., Haan C.K., Ferguson T.B., Peterson E.D. (2006). Society of Thoracic Surgeons National Cardiac Surgery Database Investigators. Bedside tool for predicting the risk of postoperative dialysis in patients undergoing cardiac surgery. Circulation.

[18] Kiers H.D., van den Boogaard M., Schoenmakers M.C., van der Hoeven J.G., van Swieten H.A., Heemskerk S., Pickkers P. (2013). Comparison and clinical suitability of eight prediction models for cardiac surgery-related acute kidney injury. Nephrol. Dial. Transplant..

[19] Arslanian S., Bacha F., Grey M., Marcus M.D., White N.H., Zeitler P. (2018). Evaluation and Management of Youth-Onset Type 2 Diabetes: A Position Statement by the American Diabetes Association. Diabetes Care.

[20] Dimitrakopoulou V.I., Tsilidis K.K., Haycock P.C., Dimou N.L., Al-Dabhani K., Martin R.M., Lewis S.J., Gunter M.J., Mondul A., Shui I.M. (2017). Circulating vitamin D concentration and risk of seven cancers: Mendelian randomisation study. BMJ.

[21] Karkouti K., Wijeysundera D.N., Yau T.M., Callum J.L., Cheng D.C., Crowther M., Dupuis J.Y., Fremes S.E., Kent B., Laflamme C. (2009). Acute kidney injury after cardiac surgery: Focus on modifiable risk factors. Circulation.

[22] Thakar C.V., Worley S., Arrigain S., Yared J.P., Paganini E.P. (2005). Influence of renal dysfunction on mortality after cardiac surgery: Modifying effect of preoperative renal function. Kidney Int..

[23] Liu Y., Davari-Farid S., Arora P., Porhomayon J., Nader N.D. (2014). Early versus late initiation of renal replacement therapy in critically ill patients with acute kidney injury after cardiac surgery: A systematic review and meta-analysis. J. Cardiothorac. Vasc. Anesth..

[24] Manche A., Casha A., Rychter J., Farrugia E., Debono M. (2008). Early dialysis in acute kidney injury after cardiac surgery. Interact. Cardiovasc. Thorac. Surg..

[25] Sugahara S., Suzuki H. (2004). Early start on continuous hemodialysis therapy improves survival rate in patients with acute renal failure following coronary bypass surgery. Hemodial. Int..

[26] Conlon P.J., Stafford-Smith M., White W.D., Newman M.F., King S., Winn M.P., Landolfo K. (1999). Acute renal failure following cardiac surgery. Nephrol. Dial. Transplant..

[27] Mangano C.M., Diamondstone L.S., Ramsay J.G., Aggarwal A., Herskowitz A., Mangano D.T. (1998). Renal dysfunction after myocardial revascularization: Risk factors, adverse outcomes, and hospital resource utilization. The Multicenter Study of Perioperative Ischemia Research Group. Ann. Intern. Med..

[28] Abel R.M., Buckley M.J., Austen W.G., Barnett G.O., Beck C.H., Fischer J.E. (1976). Etiology, incidence, and prognosis of renal failure following cardiac operations. Results of a prospective analysis of 500 consecutive patients. J. Thorac. Cardiovasc. Surg..

[29] Gailiunas P., Chawla R., Lazarus J.M., Cohn L., Sanders J., Merrill J.P. (1980). Acute renal failure following cardiac operations. J. Thorac. Cardiovasc. Surg..

[30] Ostermann M.E., Taube D., Morgan C.J., Evans T.W. (2000). Acute renal failure following cardiopulmonary bypass: A changing picture. Intensive Care Med..

[31] Andersson L.G., Ekroth R., Bratteby L.E., Hallhagen S., Wesslen O. (1993). Acute renal failure after coronary surgery—A study of incidence and risk factors in 2009 consecutive patients. Thorac. Cardiovasc. Surg..

[32] Yang X.M., Tu G.W., Gao J., Wang C.S., Zhu D.M., Shen B., Liu L., Luo Z. (2016). A comparison of preemptive versus standard renal replacement therapy for acute kidney injury after cardiac surgery. J. Surg. Res..

[33] Steinthorsdottir K.J., Kandler K., Agerlin Windelov N., Steinbruchel D.A. (2013). Renal replacement therapy after cardiac surgery; renal function recovers. Scand. Cardiovasc. J..

[34] Thongprayoon C., Cheungpasitporn W., Shah I.K., Kashyap R., Park S.J., Kashani K., Dillon J.J. (2015). Long-term Outcomes and Prognostic Factors for Patients Requiring Renal Replacement Therapy After Cardiac Surgery. Mayo Clin. Proc..

